# CIP4 is required for the hypertrophic growth of neonatal cardiac myocytes

**DOI:** 10.1186/1423-0127-20-56

**Published:** 2013-08-03

**Authors:** Francesca Rusconi, Hrishikesh Thakur, Jinliang Li, Michael S Kapiloff

**Affiliations:** 1 Cardiac Signal Transduction and Cellular Biology Laboratory, Interdisciplinary Stem Cell Institute, Departments of Pediatrics and Medicine, Leonard M. Miller School of Medicine, University of Miami, R198, P.O. Box 016960, Miami, FL 33101, USA

**Keywords:** CIP4, Heart, Hypertrophy, Myocyte

## Abstract

**Background:**

CIP4 is a scaffold protein that regulates membrane deformation and tubulation, organization of the actin cytoskeleton, endocytosis of growth factor receptors, and vesicle trafficking. Although expressed in the heart, CIP4 has not been studied with regards to its potential function in cardiac myocytes.

**Results:**

We now show using RNA interference that CIP4 expression in neonatal rat ventricular myocytes is required for the induction of non-mitotic, hypertrophic growth by the α-adrenergic agonist phenylephrine, the IL-6 cytokine leukemia inhibitor factor, and fetal bovine serum, as assayed using morphometry, immunocytochemistry for the hypertrophic marker atrial natriuretic factor and [^3^H]leucine incorporation for de novo protein synthesis. This requirement was consistent with the induction of CIP4 expression by hypertrophic stimulation. The inhibition of myocyte hypertrophy by CIP4 small interfering oligonucleotides (siRNA) was rescued by expression of a recombinant CIP4 protein, but not by a mutant lacking the N-terminal FCH domain responsible for CIP4 intracellular localization.

**Conclusions:**

These results imply that CIP4 plays a significant role in the intracellular hypertrophic signal transduction network that controls the growth of cardiac myocytes in heart disease.

## Background

Myocyte hypertrophy is a compensatory response of the heart to chronic stress whether due to volume or pressure overload. In pathologic conditions, however, cardiac hypertrophy is concomitant with alterations in contractility and energy metabolism, increased cell death, and the appearance of interstitial fibrosis that can culminate in the development of heart failure. These changes are controlled by a network of mitogen-activated protein kinase, cyclic nucleotide, calcium, and phosphoinositide-dependent intracellular signaling pathways [1]. The identification of novel strategies by which to attenuate pathologic remodeling remains an important goal of cardiac signal transduction research. As the organizers of “nodes” in the intracellular signaling network, scaffold proteins may be of interest as potential therapeutic targets [2].

**Figure 1:**
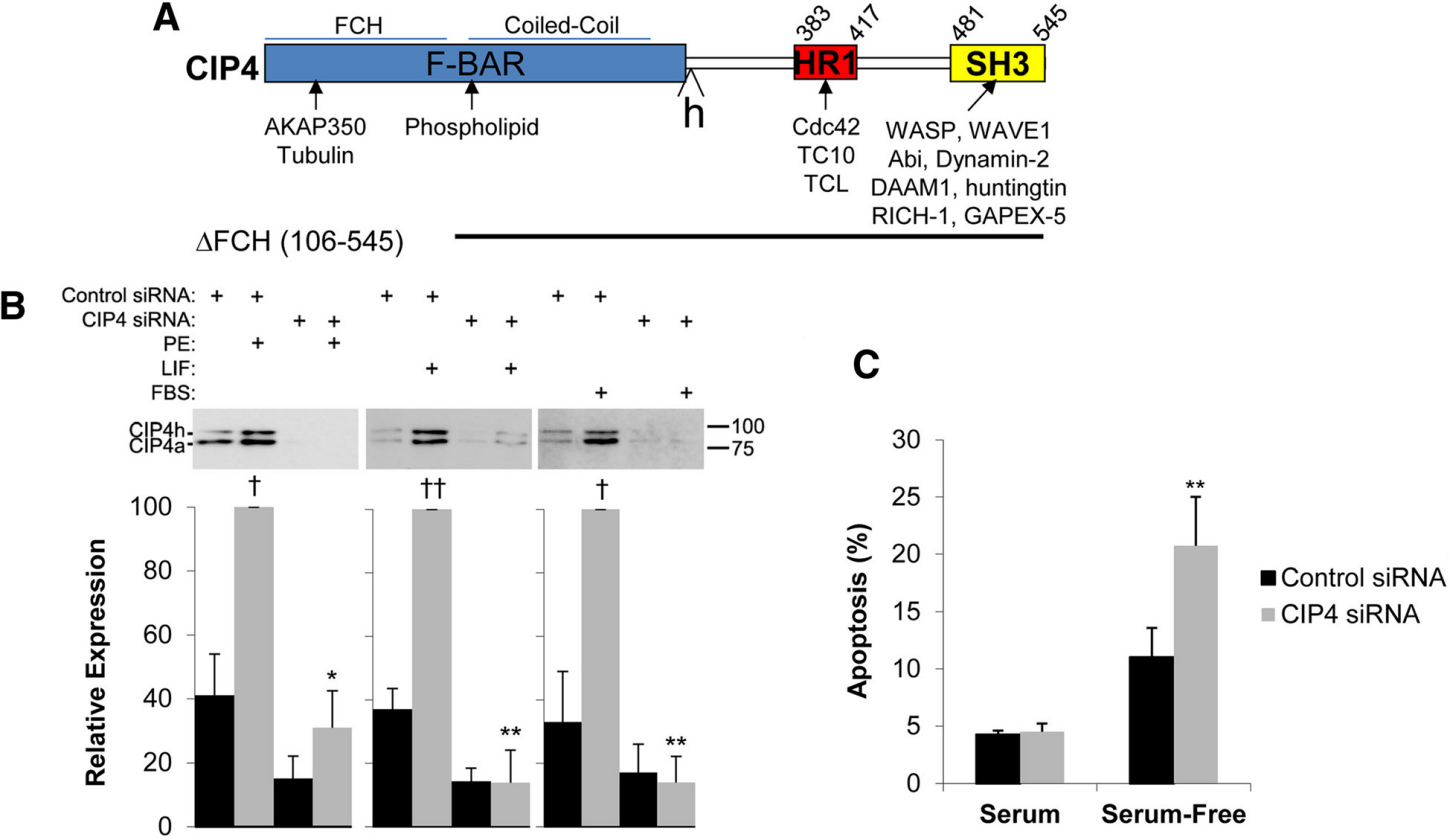
**CIP4 structure and expression. A**. Primary structure of CIP4 showing domains and binding sites for known protein partners. “h” indicates the 56 amino acid residue insertion present in CIP4h. **B**. Neonatal rat ventricular myocytes were transfected with control or CIP4 siRNA and then stimulated with 10 μM PE, 1000 U/mL LIF, or 10% FBS for two days as indicated. CIP4 proteins in whole cell lysates were detected using a mouse anti-CIP4 antibody. *n* = 4–5. ^†^*p*-values vs. no drug control; ^*^p-values vs. control siRNA-transfected myocytes treated with the same agonist. **C**. Myocytes were transfected with control or CIP4 siRNA and cultured in minimal media +/− 4% horse serum for two days before TUNEL staining. % TUNEL-positive nuclei are indicated. **p < 0.005 for CIP4 siRNA vs. control siRNA. *n* = 3–5.

CIP4 is a modular scaffold protein involved in the regulation of cellular morphology that can serve as an effector for the Rho family small GTPases Cdc42, TC10, and TCL [3]. CIP4 contains a N-terminal F-Bar (Fes-CIP4 homology [**F**CH] – **B**in/**A**mphyphysin/**R**vs) domain that binds both cytoskeletal proteins and negatively-charged membrane phospholipids, a HR1 domain that binds active, GTP-bound Rho family members, and a C-terminal SH3 (SRC Homology 3) domain that binds a variety of proteins involved in the regulation of the actin cytoskeleton and small GTPase signaling (Figure 1A). As a result, CIP4 has roles in the regulation of membrane deformation and tubulation, dynamic remodeling of the actin cytoskeleton, endocytosis, and vesicle trafficking [4,5]. CIP4 also regulates filopodial and lamellipodial protrusion, affecting the invasiveness and metastasis of cancer cells and neurite extension in neurons [6,7]. Recently, two groups described CIP4 knock-out mice that were normal in appearance and fertile. One group showed that CIP4-null embryonic fibroblasts were defective in endocytosis [4]. This was reflected by increased GLUT4 (glucose transporter 4) levels in skeletal muscle membranes and lower post-prandial glucose levels in vivo. The other group showed that despite normal development of the immune system, the CIP4-null mice had poor T-cell function [5]. These defects were related to impaired T cell migration and adhesion, presumably reflecting the importance of CIP4 to regulation of the actin cytoskeleton.

Although readily detectable in the heart, nothing has been published about the function of CIP4 and other F-Bar proteins in the cardiac myocyte. In other cell types, CIP4 has been shown to regulate epidermal growth factor receptor (EGFR) endocytosis [8]. Interestingly, EGFR transactivation has been implicated in the induction of cardiac hypertrophy by α-adrenergic, angiotensin II, and other G-protein coupled receptors [9,10]. Cardiac myocyte hypertrophy is non-mitotic growth of the cell characterized by increased myofibrillar assembly and changes in gene expression that includes a re-expression of “fetal” genes [1]. Due to CIP4’s function in regulating the actin cytoskeleton, we considered that CIP4 may serve a role in the control of cardiac myocyte hypertrophy. We now present data that CIP4 is required for the hypertrophic growth of cultured primary neonatal rat ventricular myocytes in response to diverse extracellular stimuli.

## Methods

### Antibodies

Commercially available antibodies were as follows: mouse anti-HA tag (Sigma), mouse anti-myc tag (monoclonal 4A6, Millipore), mouse anti CIP4 (BD Biosciences) , mouse anti-α-actinin (monoclonal EA-53, Sigma), rabbit anti-rat atrial natriuretic factor (ANF; US Biological), horseradish peroxidase (HRP)-conjugated donkey secondary antibodies (Jackson ImmunoResearch) and Alexa dye-conjugated donkey secondary antibodies (Molecular Probes). Rabbit anti-CIP4 antibodies OR048 and OR049 were generated using His_6_-tagged CIP4 260–513 protein purified from E. *Coli* DE3 RIL (Stratagene) containing pET30b-CIP4 260–513.

### Expression vectors and siRNA

All CIP4 expression plasmids and adenovirus were generated using a human cDNA for the full-length 4h isoform. pET30b-CIP4 260–513 was generated using a BamHI - SacI fragment of the cDNA. A myc tag was added to the N-terminus of the CIP4 open reading frame by subcloning the CIP4 cDNA into the PspOMI – Pac I sites in the pCMVTag3a mammalian expression vector (Stratagene). The myc-CIP4 ΔFCH expression plasmid was generated by deleting the Sal I – Sca I fragment in myc-CIP4 wildtype vector, deleting the region encoding the first 105 amino acid residues of CIP4h. All adenovirus were constructed by subcloning myc-tagged CIP4 cDNAs into the pTRE shuttle vector and the Adeno-X-Tet off-system (Clontech) and purified after amplification using Vivapure AdenoPACK kits (Sartorius Stedim). These adenovirus conditionally express recombinant proteins when co-infected with virus expressing tetracycline transactivator (adeno-tTA for “tet-off” or reverse tTA for “tet-on”) under the control of the CMV promoter. All plasmid constructs were verified by sequencing, and details of the various constructions (including new deletion mutants generated by oligonucleotide-based site-directed mutagenesis) are available upon request. On-target plus siRNA for CIP4 was CCAAAGAUGACCCCGAAAU (Dharmacon J-095808-11-0010). Control siRNA was Dharmacon On-Targetplus Non-targeting siRNA #1.

### Neonatal rat myocytes isolation and culture

All experiments involving animals were approved by the Institutional Animal Care and Use Committee at the University of Miami. 1–3 day old Sprague–Dawley rats were decapitated and the excised hearts placed in 1× ADS Buffer (116 mM NaCl, 20 mM HEPES, 1 mM NaH2PO4, 5.5 mM glucose, 5.4 mM KCl, 0.8 mM MgSO4, pH 7.35). The atria were carefully removed and the blood washed away. The ventricles were minced and incubated with 15 mL 1× ADS Buffer containing 3.3 mg type II collagenase (Worthington, 230 U/mg) and 9 mg Pancreatin (Sigma) at 37oC while shaking at 80 RPM. After 15 minutes, the dissociated cardiac myocytes were separated by centrifugation at 50 × *g* for 1 minute, resuspended in 4 mL horse serum and incubated 37oC with occasional agitation. The steps for enzymatic digestion and isolation of myocytes were repeated 10–12 times to maximize yield. The myocytes were pooled and spun down again at 50 × *g* for 2 minutes and resuspended in Maintenance Medium (DMEM:M199, 4:1) supplemented with 10% horse serum and 5% fetal bovine serum (FBS). To remove any contaminating fibroblasts, the cells were pre-plated for 1 hour before plating on gelatin-coated tissue culture plasticware. This procedure yields >90% pure cardiac myocytes. After 1 day in culture, the media was changed to maintenance medium containing 0.1 mM bromodeoxyuridine (BrdU) to suppress fibroblast growth.

Experiments were initiated 1 day after myocyte isolation. Adenoviral infection was performed by addition of adenovirus (multiplicity of infection = 10–100) to the media. Plasmids and siRNA oligonucleotides were transfected using Transfast (Promega) and Dharmafect 1 (Thermofisher), respectively, as recommended by the manufacturers using cells cultured in maintenance medium supplemented with 4% horse serum. Starting the day after gene transduction, the cells were treated for as long as 2 days, as indicated for each experiment with 10 μM phenylephrine (PE), 10% FBS, or 1000U/ml leukemia inhibitor factor (LIF) to induce hypertrophy.

### Immunocytochemistry

Cultured neonatal cardiomyocytes on plastic coverslips were fixed in 3.7% formaldehyde in PBS, permeabilized with 0.3% Triton X-100 in PBS, and blocked with PBS containing 0.2% BSA and 1% horse serum for 1 hour. The slides were then sequentially incubated for 1 hour with primary and Alexa fluorescent dye-conjugated specific-secondary antibodies (Invitrogen, 1:1000) diluted in blocking buffer. The slips were washed three times with blocking buffer. 1 μg/mL Hoechst 33258 was included in the last wash stop to label nuclei. Slides were sealed in SlowFade Gold antifade buffer (Invitrogen) for fluorescent microscopy. Wide-field images were acquired using a Leica DMI 6000 Microscope. Myocyte immunocytochemistry and morphometrics was performed as previously described by digital wide-field fluorescent microscopy using IPLab 4.0 software (BD Biosciences) [11]. TUNEL staining was performed using the In Situ Cell Death Detection Kit, TMR red, (Roche) as recommended by the manufacturer.

### Assay for de novo protein synthesis

Myocytes were plated in 24-well, gelatin-coated tissue culture plates at 60,000 cells per well and transfected as above with siRNA oligonucleotides. After one day in maintenance media with BrdU, the myocytes were incubated for 24 hours in maintenance media containing BrdU, 2 μCi [4,5-^3^H]leucine, and agonist as indicated. After washing with ice-cold PBS, total proteins were precipitated with 10% trichloroacetic acid, solubilized with 0.4 M sodium hydroxide and analyzed by liquid scintillation counting.

### Western blots

Western blots were developed using horseradish peroxidase-conjugated donkey secondary antibodies, Supersignal West Chemiluminescent Substrates (Thermo Scientific) and a Fujifilm LAS-3000 imaging system.

### Statistics

All data are expressed as mean ± s.e.m. Each *n* represents the results of experiments using separate primary cultures. Within each experiment, >25 cells were measured for each condition for both morphometric and ANF expression studies. ANOVA was calculated with *α* = 0.05. Post-hoc *p*-values were calculated using two-tailed, paired Student's *t*-tests. Repeated symbols are as follows: * p< 0.05; ** *p*<0.005, etc.

## Results and discussion

**Figure 2:**
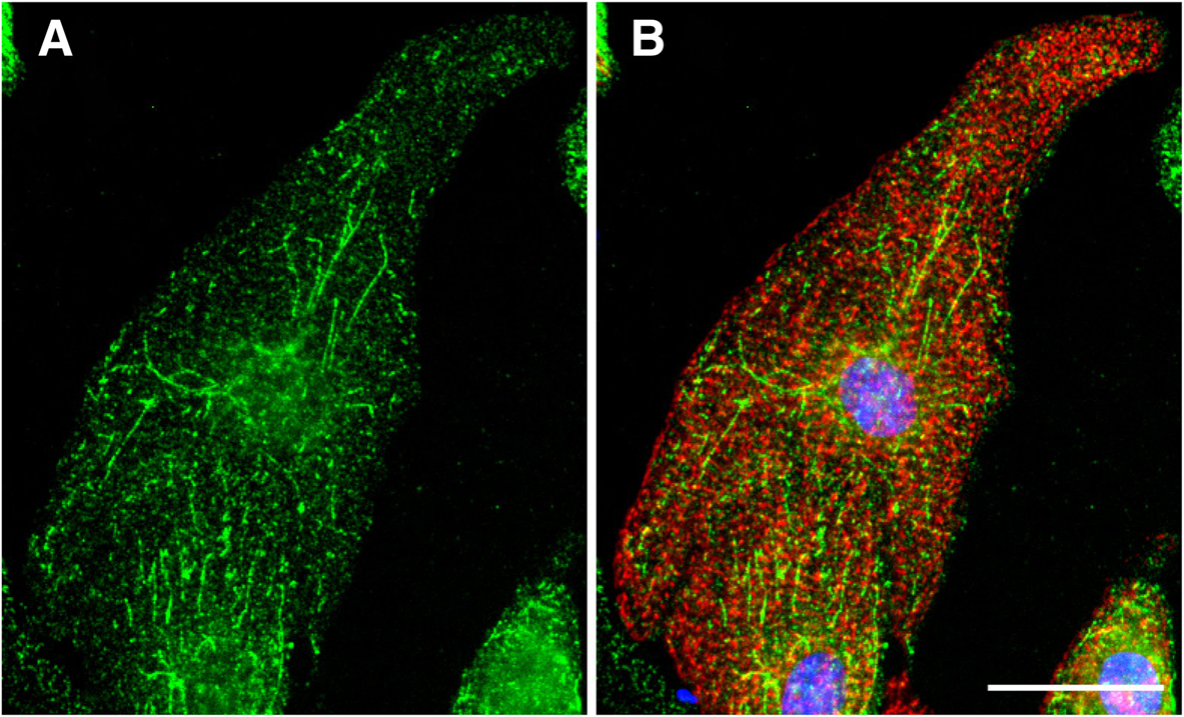
**CIP4 localization in myocytes.** Myocytes expressing myc-CIP4 WT were stained with myc antibodies (green) and α-actinin (red) antibodies and Hoechst nuclear stain (blue). Bar = 20 μm. Panel **A** shows the green channel alone. *n* > 3. Panel **B** is a composite image of myc and actinin antibody staining and the nuclear stain.

As found for adult heart tissue [4], there were two CIP4 bands detectable by western blot of neonatal rat ventricular myocyte extracts (Figure 1B). These bands represent CIP4h and CIP4a, that are identical except for a 56 amino acid residue insertion C-terminal to the F-Bar domain present in CIP4h due to alternative mRNA splicing (Figure 1A) [12]. The expression of both isoforms was consistently induced 2–3 fold by culture of the myocytes in the presence of hypertrophic agonists (Figure 1B), including by the α-adrenergic agonist phenylephrine (PE), the IL-6 cytokine leukemia inhibitory factor (LIF), and serum (FBS). We attempted to determine the localization of CIP4 in the cardiac myocyte by immunocytochemistry. However, the available CIP4 antibodies did not afford a significant signal for endogenous CIP4 protein in the myocytes, regardless of the culture condition. We were able to express myc-tagged CIP4h protein in the myocytes by adenoviral infection (Figure 2). myc-CIP4 was expressed throughout the cytosol in a reticular/punctate pattern that did not coincide with Z-line staining by an α-actinin antibody. The staining pattern was reminiscent of the CIP4 distribution in other cell types in which CIP4 is associated with microtubules, endosomes, membrane tubules and other membrane structures [8,13,14].

To test whether CIP4 was required in cardiac myocytes for hypertrophic growth, we transfected neonatal rat ventricular myocytes with control or CIP4 specific siRNA oligonucleotides (Figure 1B). Whether the cells were cultured in minimal media or in the presence of the different hypertrophic agonists, the expression of endogenous CIP4 protein was efficiently inhibited. As assayed by TUNEL staining, inhibition of CIP4 expression did not induce myocyte death when the cells were cultured in the presence of serum (Figure 1C), albeit in the absence of any growth stimulus, TUNEL staining was increased from 11% to 21% by the CIP4 siRNA. Likewise, CIP4 siRNA did not induce appreciable cell death when the myocytes were stimulated with other hypertrophic stimuli (see below).

**Figure 3:**
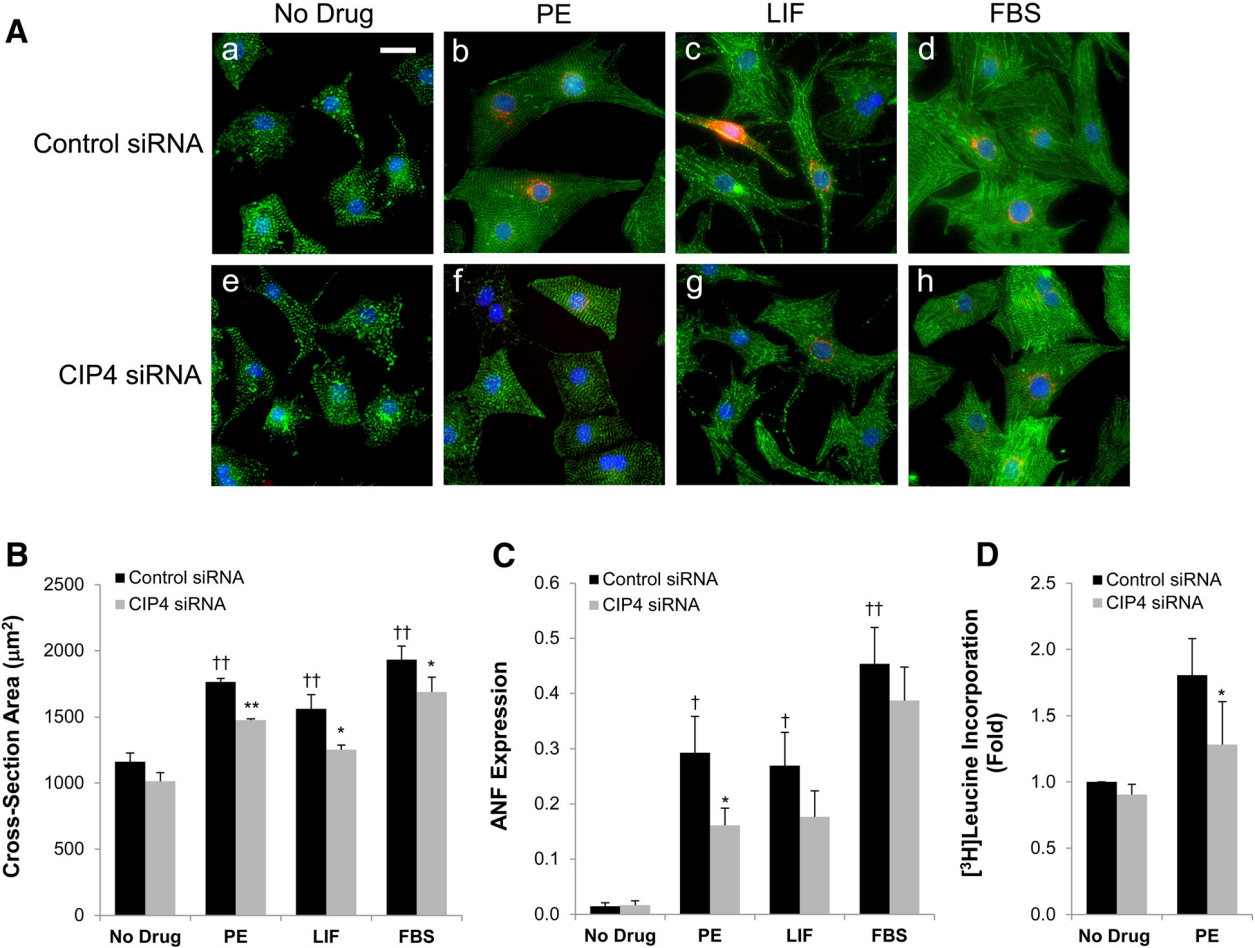
**CIP4 is important for neonatal rat ventricular myocyte hypertrophy. A**. Myocytes were transfected with control or CIP4 siRNA oligonucleotides and cultured ± 10% fetal bovine serum (FBS), 10 μM PE, or 1000 U/mL LIF. After treatment for 2 days, the myocytes were stained for α-actinin (green), ANF (red) and Hoechst (blue); bar = 20 μm. **B**. Cross-section area of myocytes in **A**. **C**. Fraction of myocytes expressing ANF in A. *n* = 5 for B and C. ANOVA (two-factor with replication): *p*-value (CIP4 siRNA vs. control siRNA) = 6 × 10^-5^**(B)** and = 0.046 **(C)**; *p*-value (between culture conditions) < 10^-7^ for both **B** and **C**. Post-hoc testing: ^*^p-values vs. control siRNA-transfected myocytes treated with the same agonist; ^†^*p*-values vs. no drug control. **D**. [3H]leucine incorporation. *n* = 2. ^*^p-value vs. PE-treated, control siRNA-transfected myocytes.

Hypertrophic myocytes are physically larger, have increased sarcomeric organization, express “fetal” genes such as that for atrial natriuretic factor (ANF), and have increased de novo protein synthesis [11,15]. Myocytes were transfected with the CIP4 or control siRNA and then cultured in the presence of PE, LIF, or FBS. After two days, cross-section area was measured using morphometric software and a qualitative assessment of sarcomeric organization was performed by α-actinin staining (Figure 3A,B). ANF expression was assayed by staining for prepro-ANF, and [^3^H]leucine incorporation was used to assay protein synthesis (Figure 3C,D). As expected, PE and FBS induced the growth in width and length of the control siRNA-transfected myocytes, while LIF, that activates the gp130-LIF cytokine receptor, induced an elongated myocyte phenotype. PE induced the most prominent sarcomeric organization as detected by α-actinin Z-line staining. All three stimuli induced ANF expression strongly. Regardless of the hypertrophic stimulus, myocytes transfected with the CIP4 siRNA were smaller in cross-section area, while not qualitatively different in overall proportion or sarcomeric organization (Figure 3A,B). Importantly, the induction of ANF expression by the hypertrophic agonists was attenuated by the CIP4 siRNA, albeit significantly only for the PE-treated cells (Figure 3C; 45%, 35%, and 15% less for PE, LIF, and FBS treated, CIP4 siRNA-transfected myocytes, respectively). To corroborate these data, we tested by [3H]leucine incorporation whether CIP4 siRNA could inhibit the de novo protein synthesis associated with PE-induced hypertrophy. Consistent with the requirement for CIP4 expression for PE-induced ANF expression, PE-stimulated [3H]leucine incorporation was significantly attenuated 48% by the CIP4 siRNA (Figure 3D). Taken together these data imply that the CIP4 scaffold contributes to signal transduction important for agonist-induced hypertrophy.

**Figure 4:**
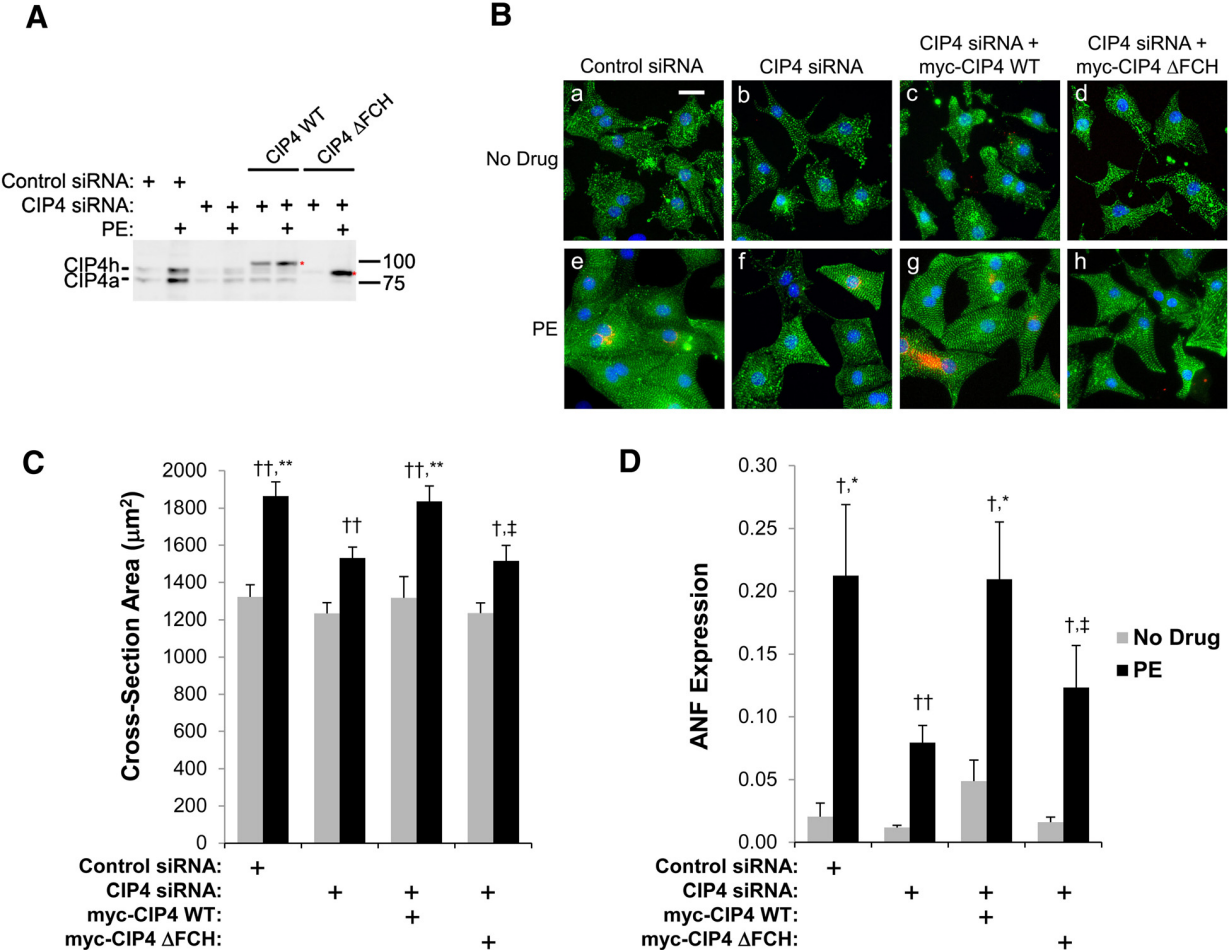
**The CIP4 FCH domain is important for neonatal rat ventricular myocyte hypertrophy.** Neonatal rat ventricular myocytes were transfected with control or CIP4 siRNA and then infected with adenovirus expressing myc-tagged CIP4 WT or ΔFCH protein. Myocytes were stimulated with 10 μM PE for two days as indicated. **A**. CIP4 proteins were detected using a mouse anti-CIP4 antibody against human CIP4 aa 411–501. (Rat and human CIP4 are 92% identical.) **B**. Immunocytochemistry for α-actinin (green), ANF (red) and Hoechst (blue); bar = 20 μm. **C**. Cross-section area of myocytes. *n* = 7. **D**. Fraction of myocytes expressing ANF. *n* = 6. ANOVA (two-factor with replication): *p*-value (among the four CIP4 expression conditions) = 0.005 **(C)** and = 0.02 **(D)**; *p*-value (± PE) < 10^-6^ for both **B** and **C**. Post-hoc testing: ^*^p-values vs. CIP4 siRNA-transfected myocytes; ^†^*p*-values comparing myocytes cultured ± PE; ^‡^*p*-values vs. myc-CIP4 WT expressing myocytes.

The requirement for CIP4 in hypertrophic growth was confirmed by rescue of CIP4 expression (Figure 4A). CIP4 siRNA or control siRNA-transfected myocytes were infected with adenovirus expressing myc-tagged wildtype (WT) CIP4 or the CIP4 ΔFCH N-terminal truncation mutant that cannot properly localize to membranes or bind microtubules [16,17]. Note that due to the PE-enhanced activity of the CMV promoter, the adenoviral-based expression of the wildtype and mutant CIP4 was higher in PE-treated cells, similar to endogenous CIP4. In this experiment (Figure 4C), the PE-induced increase in myocyte cross-section area was attenuated 42% by the CIP4 siRNA. In addition, PE-induced ANF expression was inhibited 63% by CIP4 siRNA (Figure 4D). Importantly, CIP4 siRNA-transfected myocytes infected with myc-CIP4 WT adenovirus were not different in size or ANF expression than control siRNA-transfected myocytes, both in the absence and presence of hypertrophic stimulus. Moreover, PE-stimulated, CIP4 siRNA-transfected myocytes expressing myc-CIP4 ΔFCH were significantly smaller and expressed less ANF than those rescued by myc-CIP4 WT expression, while similar in size and ANF expression to the non-rescued CIP4 siRNA-transfected myocytes (Figure 4B,C). Since the FCH domain confers CIP4 association with the plasma membrane and microtubules [16,17], this result suggests that the proper localization of the CIP4 scaffold is required to mediate hypertrophic signal transduction in cardiac myocytes.

## Conclusions

Our results demonstrate that the scaffold protein CIP4 is required for the hypertrophic growth of neonatal cardiac myocytes in response to diverse stimuli. Given the increased CIP4 expression in neonatal myocytes cultured in the presence of hypertrophic agonists, we expect that CIP4 expression will be induced during the normal post-natal maturation of the heart, if not also in cardiac myocytes subject to pathologic stress. In other cell types, CIP4 regulates membrane deformation and tubulation, the cytoskeleton, endocytosis, and vesicle trafficking [4,5]. As a result, it will be important to investigate whether in myocytes CIP4 regulates plasmalemmal receptors or signal transduction further downstream. The known association of CIP4 with growth factor receptors and regulators of the actin cytoskeleton suggests that CIP4 is important via modulation of these pathways for the physiologic hypertrophy present in development [8,14]. While the data provided by PE and LIF are consistent with an additional role for CIP4 in heart disease, experimentation using the appropriate knock-out model is required to ascertain whether CIP4 is required for pathological hypertrophy in vivo. These future studies will elucidate whether CIP4 might be a therapeutic target in the prevention of heart failure that results from pathological cardiac stress such as in pressure overload or post-myocardial infarction.

## Abbreviations

ANF: Atrial natriuretic factor; CIP4: Cdc42-interacting protein 4; FBS: Fetal bovine serum; LIF: Leukemia inhibitory factor; PE: Phenylephrine; siRNA: Small interfering RNA.

## Competing interests

The authors declare that they have no competing interests.

## Authors’ contributions

FR, HT, and JL performed the experiments. FR, JL, and MSK designed the research and wrote the manuscript. All authors read and approved the final manuscript.
