# Correction: CIP4 is required for the hypertrophic growth of neonatal cardiac myocytes

**DOI:** 10.1186/1423-0127-21-45

**Published:** 2014-06-02

**Authors:** Francesca Rusconi, Hrishikesh Thakur, Jinliang Li, Michael S Kapiloff

**Affiliations:** 1 Cardiac Signal Transduction and Cellular Biology Laboratory, Interdisciplinary Stem Cell Institute, Departments of Pediatrics and Medicine, Leonard M. Miller School of Medicine, University of Miami, R198, P.O. Box 016960, Miami, FL 33101, USA

## Correction

In the published work [1], Figure three A panels e and f and Figure four B (Figure 1B here) panels b and f represent the same types of samples in two different experiments, i.e., CIP4 siRNA-transfected myocytes cultured in the absence and presence of phenylephrine, respectively. However, in the original version of Rusconi, et al. [1], the panels in Figure three A e and f were unintentionally duplicated in the panels in Figure four B (Figure 1B here) b and f, respectively. In this correction, a new Figure four (Figure 1 here) is provided with different panels for figure four B (Figure 1B) b and f. The interpretation and conclusion of the depicted experiments remain the same.

**Figure 1:**
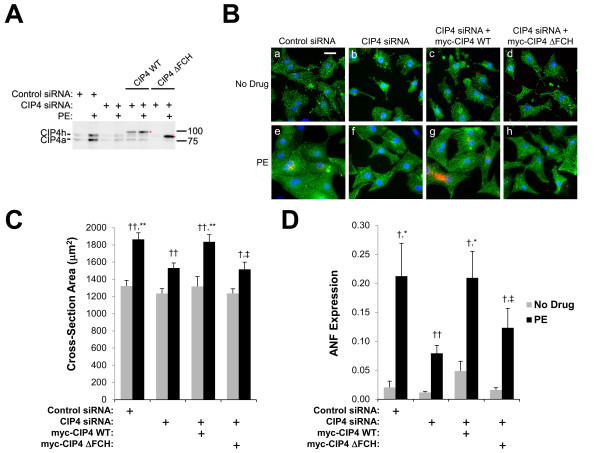
**The CIP4 FCH domain is important for neonatal rat ventricular myocyte hypertrophy.** Neonatal rat ventricular myocytes were transfected with control or CIP4 siRNA and then infected with adenovirus expressing myc-tagged CIP4 WT or ΔFCH protein. Myocytes were stimulated with 10 μM PE for two days as indicated. **A**. CIP4 proteins were detected using a mouse anti-CIP4 antibody against human CIP4 aa 411–501. (Rat and human CIP4 are 92% identical.) **B**. Immunocytochemistry for α-actinin (green), ANF (red) and Hoechst (blue); bar = 20 μm. **C**. Cross-section area of myocytes. *n* = 7. **D**. Fraction of myocytes expressing ANF. *n* = 6. ANOVA (two-factor with replication): *p*-value (among the four CIP4 expression conditions) = 0.005 (C) and = 0.02 (D); *p*-value (±PE) < 10^-6^ for both B and C. Post-hoc testing: ^*^p-values vs. CIP4 siRNA-transfected myocytes; ^†^*p*-values comparing myocytes cultured ± PE; ^‡^*p*-values vs. myc-CIP4 WT expressing myocytes.

Corrected Figure four (Figure 1 here):
